# Effects of Different Lighting Regimes on Reproductive Performance of Rabbit Does and Growth of Weaned Rabbits

**DOI:** 10.3390/ani16213397

**Published:** 2026-10-28

**Authors:** Yifei Zhang, Jiaoyue Zhu, Yingjie Wu, Yinghe Qin

**Affiliations:** State Key Laboratory of Animal Nutrition and Feeding, College of Animal Science and Technology, China Agricultural University, No. 2 Yuanmingyuan West Road, Haidian District, Beijing 100193, China

**Keywords:** lighting regime, rabbit does, weaned rabbits, reproductive performance, growth performance

## Abstract

Light is a critical environmental factor in rabbit production, yet systematic evaluation of low-light conditions for both reproduction and growth remains limited. This study assessed the effects of three combined lighting regimes: bright–dark (80 lx, 16L:8D for 6 days pre-AI plus 0–2 lx, 0L:24D during other periods), bright–dim light (80 lx, 16L:8D for 6 days pre-AI plus 4–6 lx, 24L:0D during other periods), and bright–bright light (80 lx, 16L:8D for 6 days pre-AI plus 75–85 lx, 12L:12D during other periods) on the reproductive performance of nulliparous Hyla does (*n* = 553 in total, with 185, 184, and 184 does in the bright–dark, bright–dim light, and bright–bright light groups, respectively) across three consecutive 42-day parities, and three single light regimes (dark, dim, bright) on the growth of weaned rabbits (*n* = 184 per group), over 20 days with digestion and metabolism trials. In the first parity, the bright–dark and bright–dim groups had significantly larger litter sizes and more live-born kits than the bright–bright group, whereas the average individual birth weight of live-born kits was significantly higher in the bright–bright light group than in the bright–dark group, with no significant difference between the bright–bright light and bright–dim groups. By the third parity, dim light improved total litter weight and live-born kit weight compared with bright light. Overall, the bright–dark regime showed more advantages in total and live-born litter size. For weaned rabbits, the dark regime significantly increased average daily gain and feed intake compared with the bright light regime, with higher crude protein and fiber digestibility. The dim regime showed intermediate values for several reproductive and growth outcomes. These findings provide a basis for the further evaluation of stage-specific lighting management, with welfare and behavioral outcomes requiring assessment before practical implementation.

## Simple Summary

Lighting is an important component of the housing environment for rabbits. Lighting conditions may affect breeding females and growing rabbits differently. This study compared three lighting programs involving near-dark, dim, and bright conditions on a commercial rabbit farm. Rabbit does were observed over three breeding cycles, and young rabbits were evaluated after weaning. All does received the same bright-light treatment before each insemination. Across the three breeding cycles, does under the bright–dark program had larger total and live-born litter sizes than those under the bright–bright program. The bright–dim program was associated with higher litter weights than the bright–bright program in the third cycle. After weaning, rabbits under the dark program consumed more feed and gained more weight than those under the bright program. These findings provide useful information for further evaluation of lighting programs at different production stages.

## 1. Introduction

Artificial lighting is an essential environmental factor in modern intensive animal production systems. Numerous studies have demonstrated that lighting characteristics, including light color, intensity, and photoperiod, regulate animal growth, development, and health [1,2,3,4]. An appropriate photoperiod can promote growth performance, enhance immune function, and improve overall productivity in livestock and poultry [5,6].

Among domestic animals, rabbits are a species that is sensitive to light. Previous studies have shown that photoperiod is one of the key factors determining reproductive performance in rabbits, due to its influence on sexual receptivity, kindling rate, and litter size [7]. Our previous study found that does exposed to a 16L:8D photoperiod for a short period before and after artificial insemination (AI) had significantly higher kindling rate, litter size, and litter weight at weaning than those continuously maintained under 12L:12D. Moreover, under this short 16L:8D photoperiod, light intensities of 80 lx and 120 lx elevated conception rate, kindling rate, and litter size (total and alive) compared with 40 lx [8]. These findings apply to the photostimulation period around AI and do not establish that higher light intensity is also beneficial during the maintenance period between these treatments. For this reason, commercial rabbitries typically use a 6-day photoperiod of 16L:8D with an intensity of 80 lx to induce estrus in does [9]. However, research on optimizing lighting regimes beyond this period to further enhance reproductive performance in does remains insufficient.

In addition to its role in reproduction, light has also been widely recognized as an important factor influencing growth in rabbits [10]. An early study demonstrated that continuous light exposure markedly enhanced growth performance, with significantly greater weight gain compared with darkness, indicating that light exerts a strong regulatory effect on growth [11]. Extending the photoperiod yields similar benefits. Rabbits reared under a long photoperiod (16L:8D) exhibited greater weight gain than those under a control photoperiod (12L:12D) [12]. These findings suggest that both continuous illumination and longer photoperiod promote growth in rabbits.

While extended photoperiods and higher light intensity enhance rabbit productivity, such lighting conditions conflict with the rabbit’s innate preference for dim or dark environments. This is because domestic rabbits have inherited the crepuscular and burrowing habits of their wild ancestors [13]. Notably, one study found that higher light intensities (80–100 lx) may temporarily reduce body weight gain during the early experimental period [14], suggesting an acute stress response despite long-term benefits. Furthermore, preference tests showed that does freely chose the darkest available cage [15], indicating that low-light conditions may better meet the needs of rabbits. Nevertheless, research that systematically evaluates low-light conditions (e.g., 1–20 lx) in terms of both reproduction and growth remains scarce.

Therefore, this study assessed the reproductive performance of rabbit does and the growth of weaned rabbits under three distinct lighting regimes (bright–dark, bright–dim, and bright–bright for does; dark, dim, and bright for weaned rabbits). We hypothesized that the bright–dim regime would optimize reproductive performance in does, while the dim regime would optimize growth performance in weaned rabbits. This study therefore aimed to provide production-scale evidence of the responses to these lighting regimes.

## 2. Materials and Methods

### 2.1. Animals and Experimental Design

All rabbits were obtained from Shandong Huifu Agriculture and Animal Husbandry Development Co., Ltd. (Liaocheng, Shandong, China). The experiments lasted from July to December 2018. All does were housed individually in cages, measuring 50 × 40 × 40 cm (length × width × height), whereas weaned rabbits were housed at four rabbits per cage of the same dimensions. All cages were equipped with plastic slatted flooring, nipple drinkers, and hopper feeder. Does and weaned rabbits received stage-specific commercial pelleted diets, with ad libitum access to feed and water. The composition and nutrient levels of the weaned rabbit diet are presented in Table S1. Ventilation was provided by fans, and wooden nest boxes were installed outside the cages for does approaching parturition. For both does and weaned rabbits, each lighting regime was implemented in a separate room. For the doe experiment, two digital temperature and humidity data loggers (Apresys 179-THL, Apresys (Shanghai) Precision Optoelectronics Co., Ltd., Shanghai, China) were installed at evenly spaced positions in the central area of each of the three doe rooms. Temperature and relative humidity were monitored continuously throughout the doe experiment, with readings recorded at 5 min intervals. For the doe experiment, differences in temperature and relative humidity among the three rooms did not exceed 2 °C and 5%, respectively. All other husbandry and management procedures were consistent across rooms.

**Figure 1:**
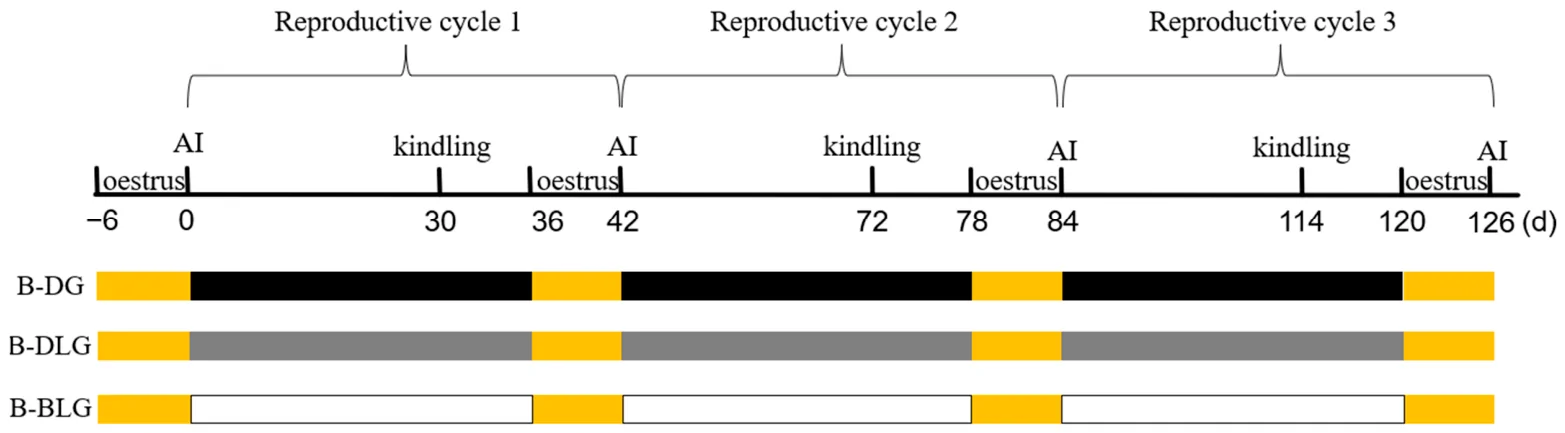
Schematic diagram of lighting schedule for does. Orange bars indicate the 6-day estrus synchronization treatment (80 lx, 16L:8D) applied to each group before each AI. Black bars indicate the dark period (0–2 lx, 0L:24D); grey bars indicate the dim light period (4–6 lx, 24L:0D); white bars indicate the bright light period (75–85 lx, 12L:12D). AI: artificial insemination. B-DG: bright–dark group. B-DLG: bright–dim light group. B-BLG: bright–bright light group.

Experiment 1: A total of 553 healthy, 6-month-old nulliparous Hyla does with similar body weight (3.69 ± 0.9 kg) were randomly divided into three groups: bright–dark, bright–dim light, and bright light, with 185, 184, and 184 does, respectively. All does were exposed to identical lighting conditions (80 lx, 16L:8D) for 6 days prior to AI to induce estrus synchronization. This pre-AI lighting procedure was routinely used at the commercial farm to trigger estrus synchronization [16]. It was applied identically to all groups as a common production-management procedure and was not an experimental contrast. Semen for AI was obtained from breeding bucks at the same farm. Semen was collected using an artificial vagina, assessed for sperm motility, pooled, and diluted before being promptly used for insemination. Each doe received 0.5 mL of diluted semen using a multidose insemination gun. Immediately after AI, each doe received an intramuscular injection of 0.5 μg of a luteinizing hormone-releasing hormone analog A3 preparation (LHRH-A3; Ningbo Sansheng Pharmaceutical Co., Ltd., Ningbo, China) in a volume of 0.5 mL to induce ovulation. After AI, the does in the dark group were subjected to near-total darkness (0–2 lx, 0L:24D) until the next synchronized estrus treatment. Does in the dim light group were exposed to a light intensity of 4–6 lx under a 24L:0D photoperiod until the next synchronized estrus treatment. Does in the bright light group were exposed to a light intensity of 75–85 lx with a photoperiod of 12L:12D until the next synchronized estrus treatment. The day of AI was designated as day 0. Each reproductive cycle lasted 42 days, and the experiment was repeated for three consecutive parities (Figure 1). Does that died or were removed from the breeding herd did not enter subsequent parities. Reasons included heat stress, postpartum death of primiparous does, escape with unconfirmed identity, and poor maternal care. Lighting conditions were controlled using dimmers and timers. Light intensity was measured using a TES-1330A digital lux meter (TES Electrical Electronic Corp., Taipei, Taiwan) to ensure consistency.

**Figure 2:**
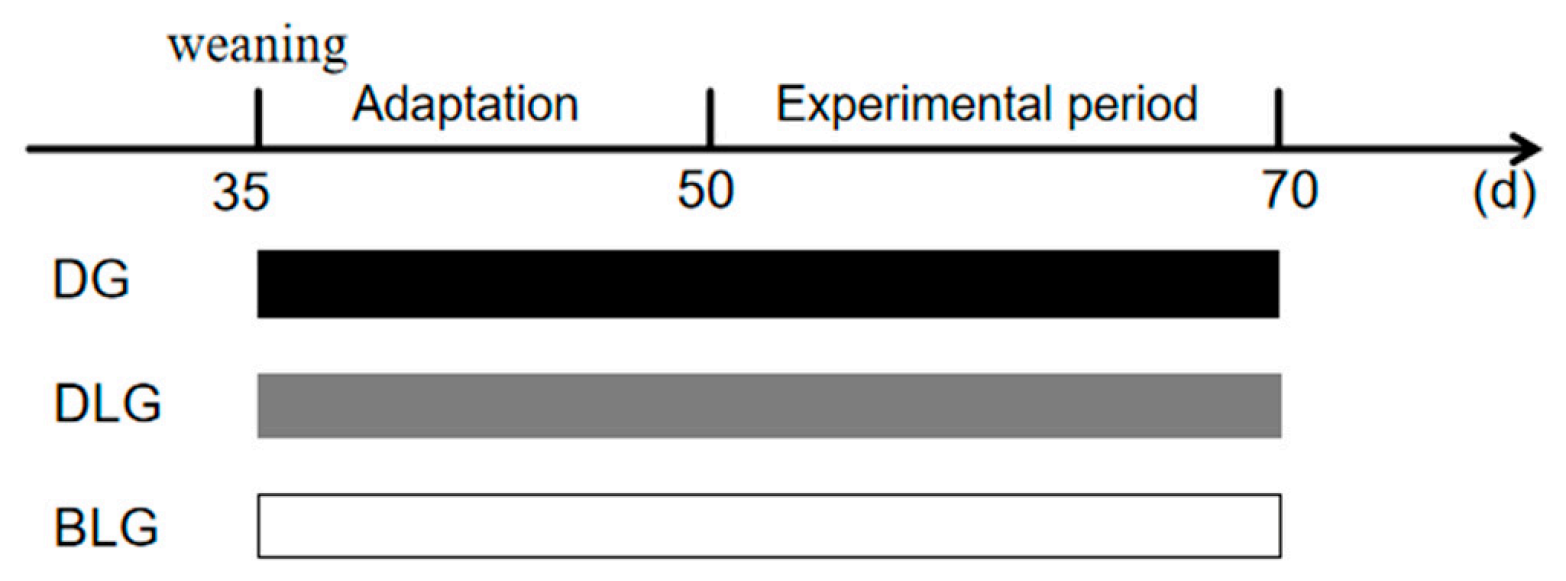
Schematic diagram of lighting schedule for weaned rabbits. Black bars indicate the dark period (0–2 lx, 0L:24D); grey bars indicate the dim light period at (4–6 lx, 24L:0D); white bars indicate the bright period of light (75–85 lx, 12L:12D). DG: dark group. DLG: dim light group. BLG: bright light group.

Experiment 2: A total of 552 weaned rabbits at 35 days of age were randomly allocated into three groups, with equal numbers of males and females (n = 184 per group). The treatment groups were defined as the dark group (0–2 lx, 0L:24D), dim light group (4–6 lx, 24L:0D), and bright light group (75–85 lx, 12L:12D) (Figure 2). Following a 15-day adaptation period, a 20-day experimental period (from 50 to 70 days of age) was conducted, during which rabbits were exposed to the respective lighting regime and their body weights and daily feed intake were recorded. During the final week of this experiment, eight rabbits with similar body weights were randomly selected using a computerized random number generator from each treatment group for the digestion and metabolism trial. The selected rabbits were individually housed in disinfected metabolic cages. Following a 3-day adaptation period, feces and urine were collected continuously for 3 days. Total feed intake, fecal output, and urine volume were recorded daily. Finally, fecal and urine samples from each rabbit over the 3 days were mixed and subsampled using the quartering method.

### 2.2. Chemical Analysis of Feed and Fecal Samples

Feed and fecal samples were analyzed according to Chinese national standard methods (GB/T). Briefly, dry matter was calculated from moisture content, which was determined by oven drying at 105 °C to constant weight (GB/T 6435-2014) [17]. Crude protein was analyzed using the Kjeldahl method (GB/T 6432-2018) [18]. Ether extract (crude fat) was determined by Soxhlet extraction (GB/T 6433-2025) [19]. Crude fiber was analyzed following GB/T 6434-2022 [20]. Ash content was determined by combustion in a muffle furnace at 550 °C.

### 2.3. Determination of Apparent Calcium Digestibility

Calcium content was determined by potassium permanganate titration according to the first method specified in Chinese national standard GB/T 6436-2018 [21]. Apparent calcium digestibility was calculated as the difference between calcium intake and fecal calcium excretion, expressed as a percentage of intake.

### 2.4. Reproductive Parameters

Pregnancy was confirmed by abdominal palpation on day 14 post-AI. The conception rate was calculated as the percentage of inseminated does that had embryos. The kindling rate was the percentage of inseminated does that successfully gave birth.

Litter size was defined as the total number of kits born per litter. Litter size of live-born kits referred to the number of kits showing signs of life at birth per litter. Total litter weight was measured immediately after kindling as the total weight of all kits in a litter after removal of placental tissues. Live-born litter weight was determined as the total weight of all live-born kits within a litter. Average individual weight of live-born kits was calculated as Average individual live-born kit weight = Live-born litter weight/Number of live-born kits.

### 2.5. Growth Performance Parameters

For the growth trial, rabbits were weighed individually at the beginning and end of the experimental period. Average daily gain was calculated as (final body weight—initial body weight)/experimental days. Average daily feed intake per weaned rabbit was calculated as (feed offered during the experiment—feed remaining at the end of the experiment)/(experimental days × 4). Feed-to-gain ratio was calculated as average daily feed intake/average daily gain.

### 2.6. Statistical Analysis

Conception rate and kindling rate were analyzed using chi-square tests. Other data were analyzed by one-way ANOVA with post-hoc Bonferroni correction. Does that died or showed signs of illness during the experiment were excluded from analysis. Statistical significance was set at *p* < 0.05. Continuous outcomes are presented as mean ± SEM, whereas counts and rates are presented as numbers and percentages, respectively.

## 3. Results

In order to explore the effects of different lighting regimes on the reproductive performance of rabbit does, the animals were subjected to dark, dim, and bright light. Their conception rate, kindling rate, and other reproductive parameters were then measured across three consecutive breeding cycles (Table 1).

In the first parity, both the bright–dark and bright–dim light groups produced significantly larger litters and more live-born kits than the bright–bright light group (*p* < 0.05), with no significant difference between the two low-light groups (*p* > 0.05). However, the average individual weight of live-born kits was significantly higher in the bright–bright light group than in the bright–dark group (*p* < 0.05); the bright–dim light group was intermediate and did not differ significantly from either. No significant differences were found among groups for conception rate, kindling rate, number of stillborn kits, total litter weight, or live-born litter weight (*p* > 0.05) (Table 2).

In the second parity, no significant differences were observed among the three groups in any reproductive indices (*p* > 0.05) (Table 3).

In the third parity, total litter weight and live-born litter weight were higher in the bright–dim light group than in the bright–bright light group (*p* < 0.05), whereas average individual live-born kit weight was higher in the bright–dim light group than in the bright–dark group (*p* < 0.05). No significant differences were found among the three groups in other reproductive parameters.

To assess the overall impact of lighting regimes on does’ reproductive performance, data from three consecutive parities were analyzed. As shown in Table 4, both total and alive litter sizes were significantly higher in the bright–dark group than in the bright–bright light group (*p* < 0.05). The bright–dim light group did not differ significantly from either group in these two measures. For average individual weight of live-born kits, the bright–bright light group outperformed the dark group (*p* < 0.05), whereas the bright–dim light group did not, again, differ significantly from either group. No significant differences were observed among the three lighting regimes in terms of conception rate, kindling rate, number of stillborn kits, or litter weight metrics (total or live).

As shown in Table 5, the average daily feed intake and average daily gain were significantly lower in the bright light group than in the dark group (*p* < 0.05), while the dim light group showed intermediate values, with no significant difference from either the dark group or the bright light group (*p* > 0.05). No significant differences were found among the three groups in the feed-to-gain ratio (*p* > 0.05).

As shown in Table 6, crude protein digestibility in weaned rabbits was significantly higher in the dark and bright light groups than in the dim light group (*p* < 0.05). Additionally, the dark group exhibited significantly higher crude fiber digestibility compared with the bright light group (*p* < 0.05), while showing no significant difference from the dim light group (*p* > 0.05). However, the apparent digestibility of calcium in the bright light group was significantly higher than that in both the dark group and the dim light group (*p* < 0.05).

## 4. Discussion

The present study compared the effects of three lighting regimes individually on the reproductive performance of rabbit does and the growth of weaned rabbits. In the does, the bright–dark regime was associated with larger litter sizes than the bright–bright regime over three consecutive parities. For weaned rabbits, those under the dark regime showed higher feed intake and average daily gain than those under the bright light regime, while the apparent digestibility of calcium was highest under the bright regime.

In wild rabbits, the breeding season generally begins as day length increases in spring and ends as it decreases toward winter [1]. Artificially extending the daily light period has been reported to improve sexual receptivity and reproductive performance in domestic rabbits [16]. Gerencsér et al. compared a constant 16L:8D photoperiod with a lighting schedule in which the photoperiod was increased from 8L:16D to 16L:8D eight days before artificial insemination (AI) in Pannon White rabbits [22]. The pre-AI extension significantly increased pregnancy rate. Zhang et al. [23] also reported that a long photoperiod (16L:8D) significantly increased the estrus rate of 5-month-old New Zealand White does at first breeding compared with 12L:12D and 8L:16D photoperiods. These findings support the use of pre-AI photoperiod extension as a biostimulation method in commercial rabbit production.

Previous findings on the effects of light intensity on the reproductive performance of rabbit does have been inconsistent. Matics et al. compared the reproductive performance of does over five consecutive parities under 10–20 lx and 150–200 lx and reported that the high-intensity group had greater numbers of live-born and weaned kits [15]. Liu et al. examined the effects of 60 lx, 80 lx, and 100 lx on the reproductive performance of Hyla rabbits and found significant differences among groups in the number of weaned kits, with the 80 lx group showing the lowest value and 60 lx or 100 lx producing the best results [24]. Ren et al. also reported that the number of weaned kits at 80 lx was significantly lower than that at 60 lx [25]. Differences among studies may be attributable to variations in lighting periods, illumination ranges, animal management, and experimental conditions. While previous studies mainly focused on illumination levels above 60 lx, the present study evaluated reproductive performance of does under staged lighting regimes that included very low-light conditions. Reproductive responses under the dim regime varied across parities, with larger litter sizes observed during the first parity and higher litter-weight traits observed during the third parity compared with the bright regime. These results extend existing observations and provide additional data for evaluating the effects of different lighting regimes on rabbit reproduction.

Similar patterns have also been reported in other livestock and poultry species. Research on laying hens indicates that the minimum light intensity required for maximum egg production is approximately 5.38 lx in environmentally controlled housing [26,27,28] and around 10 lx in open-sided houses; intensities exceeding 10 lx generally offer no additional benefits and may instead trigger abnormal behaviors such as aggression, hyperactivity, and feather pecking, thereby impairing production performance [29]. Although these findings cannot be directly extrapolated to rabbits, they indicate that increasing illumination above a species-appropriate level does not necessarily provide additional production benefits. Moreover, rabbits are crepuscular animals, with activity commonly concentrated around dawn and dusk when natural illumination is relatively low. Szendrő et al. noted that domestic rabbits exhibit behavioral traits adapted to low-light environments [7]. For example, in most experiments, does nursed their kits during the night, with a peak near midnight [30,31,32]; correspondingly, preference tests by Matics et al. demonstrated that does significantly prefer darker cage locations (10 lx) [15]. The preference of rabbits for darker environments provides a possible behavioral context for the reproductive responses observed under the dim regime. However, this preference does not establish continuous darkness as biologically optimal. For instance, although the higher feed intake and average daily gain observed under the dark regime in the present study represent favorable production outcomes, they do not indicate its suitability for long-term health or welfare.

The second-parity trial was conducted in summer (August to September), when heat stress may have partially masked the effects of lighting regime. High ambient temperature is a major factor that suppresses reproductive activity in does and disrupts normal physiological functions [33]. In the third parity, no significant differences in litter size were detected among groups, but total litter weight, litter weight of live-born kits, and average weight of live-born kits were significantly higher in the dim light group. These results indicate that the effects of lighting regimes on reproductive performance may vary across reproductive cycles. Because no physiological or behavioral measurements were conducted in the present study, the underlying causes of these differences cannot be determined and warrant further investigation.

In the growth trial, weaned rabbits in the dark group exhibited significantly higher average daily feed intake and average daily gain than those in the bright light group, with no significant difference in feed conversion ratio; these results are similar to those of Guo et al., who reported that rabbits in a 24 h dark photoperiod had significantly higher average daily feed intake and average daily gain than those in a 12L:12D regime [34]. However, these findings appear to differ from those reported in an earlier study conducted in 1928, in which continuous illumination provided by neon lights increased body weight in male rabbits compared with the sunlight group and the dark group. It should be noted that the earlier study used rabbits aged 8–10 months of unspecified breed, all of which were males, and light intensity was not measured in any of the three groups. Therefore, differences in physiological stage, sex, and lighting conditions may have contributed to the inconsistent results among studies. Furthermore, feeding behavior in rabbits is regulated by the light–dark cycle; Szendrő et al. have indicated that the peak of feed intake normally occurs during the dark phase [7]. The parallel increases in feed intake and daily gain, with no change in feed conversion ratio, suggest that the enhanced growth performance in the dark group was primarily driven by higher feed intake rather than by improved metabolic efficiency.

Li et al. found that total darkness treatment significantly improved feed conversion efficiency and intestinal barrier function in growing rabbits by activating the cecal circadian clock system and modulating the composition of the intestinal microbiota and short-chain fatty acid production [35]. This may partly explain why the dark group exhibited the highest apparent digestibility of crude protein and crude fiber in the present study. The present study also found that apparent calcium digestibility in the bright group was significantly higher than that in the dark and dim groups. However, this finding should be interpreted cautiously because of the small sample size (n = 8 per group) and substantial individual variability, and requires confirmation in a larger study. Higher apparent calcium digestibility does not directly demonstrate increased whole-body calcium retention or skeletal calcium deposition. Since no physiological parameters related to calcium regulation were evaluated in the present trial, the precise biological mechanisms underlying this difference remain unclear.

The present study provides production-scale evidence regarding reproductive, growth and digestibility outcomes under three complete lighting regimes. It should be noted that each regime consisted of a specific combination of illumination level and photoperiod. Therefore, the observed differences reflect the overall responses to the tested lighting regimes, rather than the independent effect of either lighting component. Further studies using a factorial design in which illumination level and photoperiod are independently controlled are warranted to clarify their respective and interactive effects. The findings provide a basis for the further evaluation of stage-specific lighting management. However, because each lighting regime was implemented in a separate room, room-specific effects could not be fully separated from lighting effects, and the findings should be interpreted cautiously and confirmed in future studies using replicated rooms. From animal welfare and circadian perspectives, the continuous-dark and continuous-dim-light regimes also warrant caution because neither provides a regular light–dark cycle for daily entrainment. Although feeding, water provision, and temperature management were consistent across groups, behavioral and welfare outcomes were not assessed. These aspects should be evaluated in further research before practical or routine commercial application.

A mixed-effects model with doe as a random effect and parity as a within-doe factor would have been more appropriate for the repeated measurements. However, because individual does could not be reliably identified across parities, we analyzed each parity separately using one-way ANOVA. Thus, our analyses compare treatments within each parity but do not establish whether treatment effects changed across parities. Bonferroni adjustment was applied to the post hoc pairwise comparisons among treatment groups following one-way ANOVA, separately for each outcome and parity. Given the number of comparisons, isolated significant findings should be interpreted cautiously.

## 5. Conclusions

In rabbit does, the bright–dark regime was associated with larger total and live-born litter sizes than the bright–bright regime over the three parities, while in weaned rabbits, the dark regime was associated with higher feed intake and average daily gain than the bright regime. These findings indicate that productive responses to lighting regimes vary with physiological stage and may provide a basis for the further evaluation of stage-specific lighting management in rabbit production.

## Figures and Tables

**Table 1:** Effects of three lighting regimes on reproductive performance of rabbit does in the first parity.

Parameters of Reproductive Performance	Bright–Dark Group	Bright–Dim Light Group	Bright–Bright Light Group	*p* Value
No. of AI	185	184	184	
Conception rate (%)	85.94 ^a^	83.15 ^a^	84.78 ^a^	0.757
Kindling rate (%)	71.89 ^a^	78.26 ^a^	73.91 ^a^	0.356
Litter size	8.10 ± 0.24 ^a^	7.96 ± 0.25 ^a^	7.02 ± 0.23 ^b^	0.003
Litter size of live-born kits	7.25 ± 0.27 ^a^	7.28 ± 0.26 ^a^	6.20 ± 0.22 ^b^	0.003
Number of stillborn kits	0.86 ± 0.12	0.68 ± 0.13	0.82 ± 0.15	0.625
Total litter weight at birth (g)	414.51 ± 11.16	424.93 ± 10.51	396.69 ± 10.65	0.170
Litter weight of live-born kits (g)	377.37 ± 12.78	389.03 ± 12.34	355.74 ± 11.50	0.146
Average individual weight of live-born kits (g)	52.48 ± 1.14 ^b^	54.09 ± 1.40 ^ab^	57.84 ± 1.28 ^a^	0.012

^ab^ Values not sharing a common superscript letter within the same row are significantly different (*p* < 0.05).

**Table 2:** Effects of three lighting regimes on the reproductive performance of rabbit does in the second parity.

Parameters of Reproductive Performance	Bright–Dark Group	Bright–Dim Light Group	Bright–Bright Light Group	*p* Value
No. of AI	146	151	148	
Conception rate (%)	80.82 ^a^	81.46 ^a^	75.68 ^a^	0.403
Kindling rate (%)	68.49 ^a^	66.89 ^a^	68.92 ^a^	0.923
Litter size	8.81 ± 0.30	8.32 ± 0.33	8.28 ± 0.33	0.437
Litter size of live-born kits	8.32 ± 0.32	7.85 ± 0.35	7.61 ± 0.37	0.337
Number of stillborn kits	0.49 ± 0.13	0.46 ± 0.15	0.68 ± 0.17	0.554
Total litter weight at birth (g)	519.10 ± 14.67	512.87 ± 16.63	513.24 ± 17.08	0.955
Litter weight of live-born kits (g)	497.00 ± 16.09	486.14 ± 17.62	479.51 ± 20.09	0.787
Average individual weight of live-born kits (g)	61.19 ± 1.43	64.31 ± 1.58	62.86 ± 2.04	0.434

^a^ Values not sharing a common superscript letter within the same row are significantly different (*p* < 0.05).

**Table 3:** Effects of three lighting regimes on reproductive performance of rabbit does in the third parity.

Parameters of Reproductive Performance	Bright–Dark Group	Bright–Dim Light Group	Bright–Bright Light Group	*p* Value
No. of AI	103	101	104	
Conception rate (%)	88.35 ^a^	84.16 ^a^	86.54 ^a^	0.683
Kindling rate (%)	78.64 ^a^	83.17 ^a^	81.73 ^a^	0.699
Litter size	11.44 ± 0.29	11.08 ± 0.35	10.58 ± 0.32	0.161
Litter size of live-born kits	10.58 ± 0.38	10.24 ± 0.39	9.70 ± 0.36	0.247
Number of stillborn kits	0.86 ± 0.21	0.84 ± 0.16	0.88 ± 0.22	0.991
Total litter weight at birth (g)	651.70 ± 16.49 ^ab^	694.40 ± 17.94 ^a^	617.09 ± 14.85 ^b^	0.004
Litter weight of live-born kits (g)	614.91 ± 20.81 ^ab^	651.13 ± 21.36 ^a^	580.52 ± 18.71 ^b^	0.048
Average individual weight of live-born kits (g)	57.67 ± 1.47 ^b^	63.63 ± 1.49 ^a^	60.92 ± 2.01 ^ab^	0.048

^ab^ Values not sharing a common superscript letter within the same row are significantly different (*p* < 0.05).

**Table 4:** Summary of the three consecutive parities.

Parameters of Reproductive Performance	Bright–Dark Group	Bright–Dim Light Group	Bright–Bright Light Group	*p* Value
No. of AI	434	436	436	
Conception rate (%)	84.79 ^a^	82.80 ^a^	82.11 ^a^	0.871
Kindling rate (%)	72.35 ^a^	75.46 ^a^	74.08 ^a^	0.924
Litter size	9.19 ± 0.18 ^a^	8.87 ± 0.19 ^ab^	8.36 ± 0.18 ^b^	0.006
Litter size of live-born kits	8.45 ± 0.20 ^a^	8.21 ± 0.20 ^ab^	7.57 ± 0.19 ^b^	0.005
Number of stillborn kits	0.74 ± 0.09	0.66 ± 0.08	0.79 ± 0.10	0.572
Total litter weight at birth (g)	509.01 ± 9.53	520.73 ± 10.18	491.88 ± 9.44	0.106
Total litter weight of live-born kits (g)	476.74 ± 10.64	485.76 ± 11.02	454.37 ± 10.66	0.104
Average individual weight of live-born kits (g)	56.59 ± 0.79 ^b^	59.66 ± 0.91 ^ab^	60.24 ± 1.00 ^a^	0.01

^ab^ Values not sharing a common superscript letter within the same row are significantly different (*p* < 0.05).

**Table 5:** Effects of three lighting regimes on growth performance of weaned rabbits.

Item	Dark Group	Dim Light Group	Bright Light Group	*p* Value
No. of rabbits	184	184	184	
Initial body weight (g)	1318.64 ± 14.36 ^a^	1290.21 ± 14.62 ^a^	1308.38 ± 12.58 ^a^	0.344
Average daily feed intake (g/d)	210.95 ± 2.43 ^a^	204.45 ± 3.23 ^ab^	199.11 ± 3.19 ^b^	0.024
Average daily gain (g/d)	39.18 ± 0.67 ^a^	37.63 ± 0.68 ^ab^	35.69 ± 0.83 ^b^	0.005
Feed/gain ratio	5.42 ± 0.07 ^a^	5.46 ± 0.08 ^a^	5.63 ± 0.09 ^a^	0.165

^ab^ Values not sharing a common superscript letter within the same row are significantly different (*p* < 0.05).

**Table 6:** Effects of three lighting regimes on the apparent total tract digestibility in weaned rabbits (n = 8, %).

Item (%)	Dark Group	Dim Light Group	Bright Light Group
Dry matter	53.76 ± 0.90	54.08 ± 1.23	52.14 ± 0.48
Crude protein	70.27 ± 1.69 ^a^	62.80 ± 2.24 ^b^	69.02 ± 0.74 ^a^
Ether extract	86.28 ± 1.29	88.17 ± 0.77	86.08 ± 0.56
Crude fiber	34.32 ± 1.12 ^a^	34.73 ± 2.18 ^ab^	29.53 ± 0.96 ^b^
Ash content	31.38 ± 1.98	32.30 ± 2.42	32.76 ± 2.98
Apparent calcium digestibility	33.77 ± 5.28 ^b^	23.07 ± 4.63 ^b^	47.72 ± 1.00 ^a^

^ab^ Values not sharing a common superscript letter within the same row are significantly different (*p* < 0.05).

## Data Availability

All the datasets in this study can be provided upon reasonable request.

## Supplementary Materials

The following supporting information can be downloaded at https://www.mdpi.com/article/10.3390/ani16213397/s1, Table S1: Composition and nutrient levels of the experimental diet (air-dry basis, %).
