# Supplementary material for: Effects of Different Lighting Regimes on Reproductive Performance of Rabbit Does and Growth of Weaned Rabbits
Source: Animals (Basel). 2026 Oct 28;16(21):3397. doi: 10.3390/ani16213397 (PMC13762210; doi:10.3390/ani16213397)
Supplement: Supplementary file 1 [file animals-16-03397-s001.zip › animals-4522939-supplementary.pdf]

Supplementary Materials

Table S1: Composition and nutrient levels of the experimental diet (air-dry basis，%)

Items	Content	Items	Content
Ingredients		Nutrient levels	
Corn	5.00	Digestible energy (MJ/kg)	16.95
Barley husk	7.00	Dry matter	90.73
Wheat shorts	5.00	Crude protein	15.68
Wheat bran	15.00	Ash contain	8.37
Soya bean stem meal	3.00	Ether extract	1.95
Peanut shell	5.00	Crude fiber	20.68
Soybean oil	0.50	Calcium	14.41
Soybean meal	8.00	Phosphorus	0.32
White powder	10.00		
Black brewer's grains	10.00		
Germ dregs	6.00		
Malt sprout flour	5.50		
Peanut stem meal	10.00		
Husk powder	5.00		
Premix	5.00		
Total	100.00		

Note: All nutrient levels are analyzed values
